# Comparative Metabolomics Study of *Chaenomeles speciosa* (Sweet) Nakai from Different Geographical Regions

**DOI:** 10.3390/foods11071019

**Published:** 2022-03-31

**Authors:** Yubo Ma, Jihan Li, Jingling Li, Li Yang, Guanle Wu, Shiyao Liu

**Affiliations:** 1 Department of Botany, College of Horticulture and Landscape Architecture, Southwest University, Chongqing 400715, China; myb19970223@email.swu.edu.cn (Y.M.); Lijingling1997@163.com (J.L.); gosicked@email.swu.edu.cn (J.L.); yl19970118@email.swu.edu.cn (L.Y.); 2 School of Life Sciences, Chongqing University, Chongqing 400044, China; 201926021021@cqu.edu.cn

**Keywords:** comparative metabolomics, widely targeted metabolomics analysis, *Chaenomeles speciosa* (Sweet) Nakai, Traditional Chinese Medicine Systems Pharmacology Database and Analysis platform, key active metabolites

## Abstract

*Chaenomeles speciosa* (Sweet) Nakai (*C. speciosa*) is not only a Chinese herbal medicine but also a functional food widely planted in China. Its fruits are used to treat many diseases or can be processed into food products. This study aims to find key metabolic components, distinguish the differences between geographical regions and find more medicinal and edible values of *C. speciosa* fruits. We used ultra-high-performance liquid chromatography–tandem mass spectrometry (UHPLC-MS/MS) and widely targeted metabolomics analysis to reveal key and differential metabolites. We identified 974 metabolites and screened 548 differential metabolites from 8 regions. We selected significantly high-content differential metabolites to visualize a regional biomarker map. Comparative analysis showed Yunnan had the highest content of total flavonoids, the highest amounts of compounds related to disease resistance and drug targets and the most significant difference from the other regions according to the Traditional Chinese Medicine Systems Pharmacology Database and Analysis Platform database, a unique platform for studying the systematic pharmacology of Chinese herbal medicine and capturing the relationship between drugs, targets and diseases. We used oral bioavailability (OB) ≥ 30% and drug likeness (DL) ≥ 0.18 as the selection criteria and found 101 key active metabolites, which suggests that *C. speciosa* fruits were rich in healthy metabolites. These results provide valuable information for the development of *C. speciosa*.

## 1. Introduction

*Chaenomeles speciosa* (Sweet) Nakai (*C. speciosa*), belonging to the Rosaceae family, is a native temperate plant widely cultivated in China, Burma, Thailand, Korea and Japan. It is distributed in Yunnan, Guizhou, Shandong, Sichuan, Zhejiang and Chongqing and widely cultivated in Hubei and Anhui provinces. These eight regions are the main production areas in China. Anhui and Hubei are the main supply areas of medicinal herbs. *C. speciosa* fruits are mainly used in traditional Chinese medicine and functional food industries such as fruit wine, fruit vinegar, preserved fruit, well-received canned food, juice and so on [1]. Currently, they are extensively applied to other fields, including the city afforestation industry, health industry and pharmaceuticals industry. More and more new varieties have been screened in recent decades.

Previous studies have confirmed that *C. speciosa* fruits have many antioxidants and different phytochemicals for anticancer [2], antioxidant [3], antiviral [4], antibacterial properties [5,6], anti-inflammation [4], antihyperlipidemic [3], antihyperglycemic [3] and anti-Parkinson properties [7]. In addition, the Pharmacopoeia of the People’s Republic of China contains two triterpenoid acids, namely oleanolic acid and ursolic acid, which are legal compounds. Moreover, many minor but active components such as catechin, chlorogenic acid, epicatechin, protocatechuic acid, caffeic acid, gallic acid and syringic acid have also been extracted from *C. speciosa* [8,9]. 

Metabolomics focuses on the measurement of metabolites and identifies changes in metabolites as a result of genetic, environmental or dietary factors [10,11]. The presence of comprehensive metabolite profiles not only helps us identify the geographical region of *C. speciosa* but also assists in learning how these factors influence the nutritional values, quality and flavor of their fruits [12]. 

*C. speciosa* metabolites have been analyzed by gas chromatography–mass spectrometry (GC-MS) [9], column chromatography (CC) [13], liquid chromatography–mass spectrometry (LC-MS) [5] and high-performance liquid chromatography (HPLC) [8] in recent decades. Previous researchers primarily focused on organic acids, triterpenes, phenolic acids, flavonoids, coumarins, triterpenoids saponins, free amino acids, essential oils and alkaloids [14], which have a comparatively high content in fruits and are relatively easy to detect. 

However, there is a lack of published reports illustrating other compounds containing phytohormones, phenylpropanoids, vitamins, carbohydrates, alcohols and so on. Meanwhile, many previous studies used high-temperature or sun-dried samples as experimental materials [5,9], which could cause the loss of active ingredients that are not heat resistant. Moreover, because the relatively old mass spectrometry database is relatively old, there are very few types of substances to detect. Until now, no study in the literature has been able to comprehensively describe the types and contents of *C. speciosa* compounds. Furthermore, previous studies on geographical regions involved very few places and did not provide detailed location information, making it impossible to trace the source [9].

## 2. Materials and Methods

### 2.1. Plant Materials and Treatment

**Figure 1:**
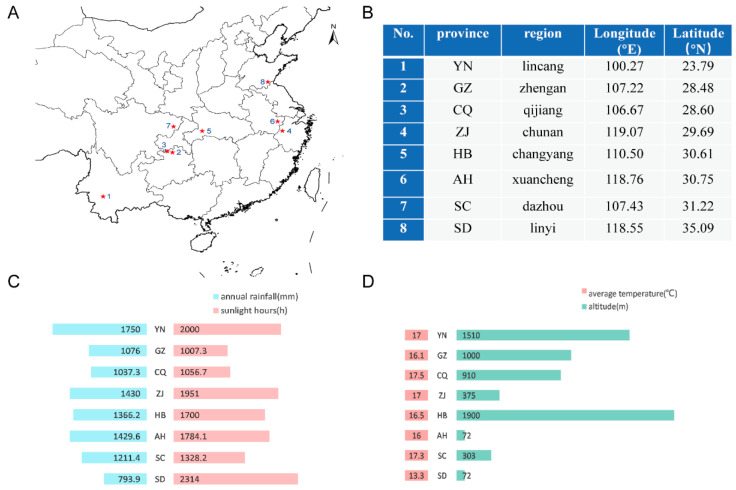
Geographical distribution and climatic conditions of *C. speciosa* samples collected. (**A**) Place of sample production. (**B**) Eight regional coordinates. (**C**) Annual rainfall (mm) and annual sunshine time (h). (**D**) Average yearly temperature (°C) and altitude (m). Climatic data were collected from the book “Prepared by the Ministry of Civil Affairs of the People’s Republic of China”; Chief Editor Li Liguo. The Political District Dictionary of the People’s Republic of China. [M] Beijing: China Social Publishing House, April 2016.

The fruits of *C. speciosa* were harvested during the mature period from eight different provinces (Figure 1) of China from 15 July to 1 August 2021. These environmental parameters for the geographical locations of the selected samples are summarized in Figure 1. Samples were collected according to the principle of representativeness, and three different sampling points were selected for each region. The selected fruits were local original wild species, and the artificial breeding of new varieties was not selected. The fresh fruits of *C speciosa.* were randomly mixed together after random collection. All of the fruits were well packed, stored at 4 °C and sent to the laboratory by air. In the laboratory, we chose fruits that were uniform in size, disease and pest free and free from mechanical damage. Then, we washed them with distilled water, deseeded them, cut them in half (cross-section) and mixed two different fruit halves (containing endocarp, exocarp and pulp) into one sample. We cut them into 2 cm small pieces that included epicarp and endocarp, placed them into liquid nitrogen and collected them with centrifuge tubes. Each group contained three replicates, and every repeat contained six different individual fruits. All of the fruits were frozen with liquid nitrogen and stored at −80 °C in preparation for the following experiments.

### 2.2. Fruit Dimensions

Fruits were selected, and the length (cm), long diameter (cm) and short diameter (cm) were measured with a Vernier caliper (±0.1 mm). Single fruit weight (g) was measured with a balance. First, the fruits were weighed, and the distance from the end of the fruit handle close to the fruit to the tail of the fruit was measured, which is the length. When placed horizontally, the distance of the wider part of the fruit, which is the long diameter, was measured, as well as the distance from the vertical section of the fruit to the desktop, which is the short diameter. ANOVA was used to calculate significant differences in fruit dimensions across the eight producing areas.

### 2.3. Metabolite Extraction

The freeze-dried samples were crushed with a mixer mill for 30 s at 60 Hz. A 50 mg amount of powder of individual samples was accurately weighed and transferred into an Eppendorf tube, followed by the addition of 700 μL of extract solution (methanol/water = 3:1, precooled at −40 °C) containing internal standard (2-chloro-DL-phenylalanine, 1 μg/mL). After vortexing for 30 s, the samples were homogenized in a ball mill at 35 Hz for 4 min and sonicated for 5 min in an ice-water bath. Homogenization and ultrasonic treatments were repeated twice. After centrifugation at 12,000 rpm for 15 min at 4 °C, the extract was absorbed and filtrated through a 0.22 μm microporous membrane. Supernatants were diluted 15 times with a methanol/water mixture (*v*:*v* = 3:1, containing internal standard), vortexed for 30 s and transferred into 2 mL glass vials. From each sample, 20 μL was taken and pooled as quality control (QC) samples. They were stored at −80 °C prior to UPLC-MS/MS analysis [15,16].

### 2.4. UHPLC Conditions

The obtained extract was analyzed by a UPLC-ESI-MS/MS system (HPLC, Shim-pack UFLC SHIMADZU CBM30A system; MS, Applied Biosystems 6500+ Q TRAP). Each sample was separated with an ACQUITY UPLC HSS T3 C18 column (Waters, 1.8 µm, 2.1 mm × 100 mm). The mobile phase consisted of solvent A (water) (0.1% acetic acid) and solvent B (acetonitrile). Gradient program: 0–0.5 min 98% A, 0.5–10 min 50% A, 10–13.1 min 5% A, 13.1–15 min 5% A; flow rate, 0.40 mL/min; temperature, 40 °C; injection volume, 2 μL.

### 2.5. ESI-Q TRAP-MS/MS Conditions

The sample composition was analyzed with a mass spectrometer (Agilent, Santa Clara, CA, USA), which was installed with a triple quadrupole (QqQ) linear ion trap (LIT) equipped with an ESI interface, operated in multiple reaction monitoring (MRM) mode and carried out with a positive/negative pattern. ESI was executed according to the following parameters: ion spray voltage, +5500/−4500 V; declustering potential, ±100 V; source temperature, 400 °C; ion curtain gas, source gas I and source gas II, 35 psi, 1:60 psi, 2:60 psi, respectively. QqQ scan was performed using multiple reaction monitoring (MRM), and the collision gas (nitrogen) was set to 5 psi. To achieve the successful transfer of a single MRM, declustering potential (DP) and collision energy (CE) were further optimized. In each cycle, a specific set of MRM transitions was monitored based on the metabolites eluted [17].

### 2.6. TCMSP Database

We matched all fruit metabolites in the Traditional Chinese Medicine Systems Pharmacology Database and Analysis Platform (TCMSP) database according to CAS number, compound name, molecular weight and structures [18]. We also acquired related targets and disease data of matched metabolites from the TCMSP database. We added the oral bioavailability (OB) parameters ≥ 30% and drug likeness (DL) ≥ 0.18 to help us screen potential key active metabolites.

### 2.7. Statistical Analysis

SCIEX Analyst Work Station Software (Version 1.6.3) was employed for MRM data acquisition and processing. The primary and secondary MS data were qualitatively assessed by searching the internal apparatus database and using a self-compiled database (Shanghai Biotree Biotech Co., Ltd., Shanghai, China). Without other special treatment, data normalization was performed by “normalization by sum”, “log transformation” and “UV scaling”. Metabolites from 28 samples (24 region samples and 4 QC samples) were analyzed by principal component analysis (PCA), orthogonal partial least squares discriminant analysis (OPLS-DA), hierarchical clustering analysis (HCA) and Pearson correlation coefficient (PCC) by R software (www.r-project.org, accessed on 20 February 2022) or SIMCA (V16.0.2, Sartorius Stedim Data Analytics AB, Umea, Sweden). In this study, the KEGG Pathway database was used to perform metabolite set enrichment analysis (MSEA) (KEGG database: http://www.genome.jp/kegg/, accessed on 20 February 2022) [19]. Metabolite relative contents were used as subjects for the analyzed differential metabolites, which had two screening standards: variable importance in projection (VIP) value > 1 and fold change (FC) < 0.5 or >2. Analysis of the significant difference in fruit dimensions was performed by ANOVA (Duncan test and least important difference method) using Statistical Product and Service Solutions (SPSS) 23.0 (IBM, Armonk, NY, USA). TBtools [20] and R software were used to plot.

## 3. Results and Discussion

### 3.1. Climatic Conditions of Geographical Regions and Effects on Fruit Dimensions of C. speciosa

Ecological factors usually influence the composition of metabolites. Different geographical regions often have diverse climatic conditions, and many other species have been reported. Wang et al. [21] investigated three typical regions of *Lycium barbarum* fruits to illustrate the effect of climate on fruit quality. Bokulich et al. [22] discriminated the grape growing areas and vineyards in Napa County and Sonoma County from California by using grape microbiota and wine metabolites [22]. Taveira et al. [23] investigated metabolic profiles to discriminate the genotypes of coffee from various regions. Similarly, the method of distinguishing origin by metabolomics has also been applied to diverse fields, such as sea cucumber [24], rice [25], dry-cured hams [26], beef [12] and others [27,28,29]. Many researchers have reviewed the effect of producing area on fruit quality [30,31,32].

We collected *C. speciosa* fruits from eight typical geographical regions, including YN (Matai, Lincang, Yunnan, China), GZ (Tongziping, Zhengan, Guizhou, China), CQ (Datong, Qijiang, Chongqing, China), ZJ (Zuokou, Chunan, Zhejiang, China), HB (Langping, Changyang, Tujia Autonomous County, Hubei, China), AH (Cintian, Xuancheng, Anhui, China), SC (Fuxing, Dazhou, Sichuan, China) and SD (Tanghe, Linyi, Shandong, China). Figure 1A shows the eight gathering points in this study, Figure 1B shows the longitude and latitude of the eight producing regions and Figure 1C,D shows the various climatic parameters. For the average temperature, SD has the lowest temperature among the eight producing areas, with temperatures ranging from 16 to 17.5 degrees in other places. As for the altitude, HB is the highest, YN is second and SD and AH are the lowest. Regarding annual rainfall and annual sunlight hours, YN is characterized by the highest annual rainfall and second lighting time, and SD is characterized by the longest lighting time and the least yearly rainfall. The yearly rainfall and lighting time of GZ and CQ are lower than those of ZJ, HB, AH and SC. Overall, we can see that the climatic conditions from these eight *C. speciosa* fruits planting zones are distinctly different.

During the commercial fruit maturing period, fruit dimensions were investigated under the same conditions. Table 1 shows the results of the analysis of variance and Duncan test. Overall, fruits from SC and GZ were obviously thicker, longer and heavier than other areas, and the most miniature fruits came from AH. The morphologies of the *C. speciosa* fruits, particularly GZ, SC and AH, were evidently different. The results mentioned above indicate that the climatic conditions of the geographical regions have essential effects on *C. speciosa* fruit size.

### 3.2. Overall Metabolites Analysis and Multivariate Analysis in C. speciosa Fruits of Different Regions

**Figure 2:**
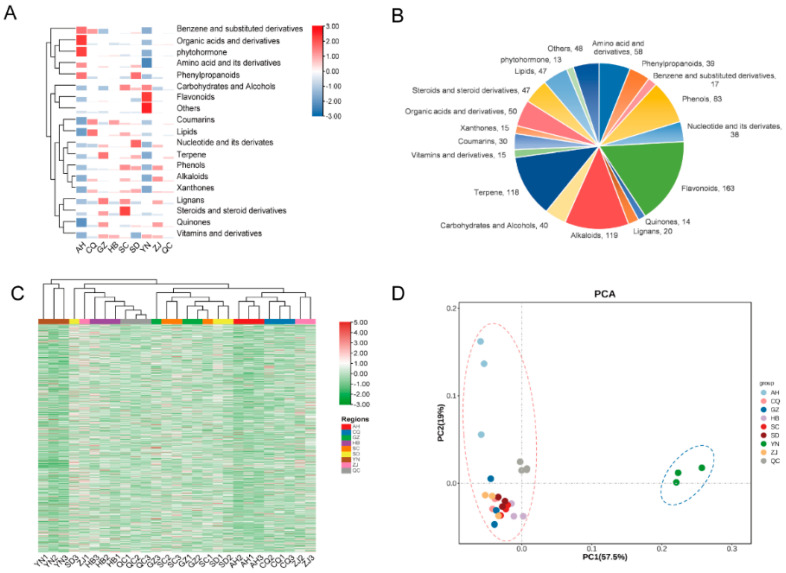
Heat map, pie graph, HCA and PCA, revealing the metabolic characteristics of *C. speciosa* fruits from eight regions. (**A**) The 974 metabolites were divided into 19 classifications, and the average value of these 19 classifications were related to 8 producing areas. (**B**) The 974 metabolites were divided into 19 classifications, and the number of compounds contained in each classification. (**C**) HCA analysis of 974 metabolites from 24 region samples and 3 QC samples. (**D**) PCA analysis of 974 metabolites from 24 region samples and 3 QC samples. QC samples represent the mixture of *C. speciosa* fruit extracts.

To further identify and better understand the metabolite differences of *C. speciosa* fruits, we performed UPLC-QqQ-MS/MS analysis of *C. speciosa* fresh fruits from the eight regions. Ultimately, we identified 974 metabolites (Table S1) and grouped them into 19 classes (Figure 2B), including 163 flavonoids, 119 alkaloids, 118 terpenes, 83 phenols, 58 amino acid and derivatives, 50 organic acids and derivatives, 47 lipids, 47 steroids and derivatives, 40 carbohydrates and alcohols, 39 phenylpropanoids, 38 nucleotides and derivates, 30 coumarins, 20 lignans, 17 benzene and substituted derivatives, 15 xanthones, 15 vitamins and derivatives, 14 quinones, 13 phytohormone and 48 other metabolites. These results indicate that UPLC-QqQ-MS/MS, with widely targeted metabolomics analysis, is a valuable and effective method for the extensive detection and identification of metabolites in plants. The results of metabolites suggest that *C. speciosa* fruits were an excellent source of flavonoids, alkaloids, terpenoids and phenols metabolites. In this study, the numbers of metabolites were greater than in previous research results, which suggests that in previous decades, metabolites were mainly detected and identified by using GC-MS, CC, LC-MS and HPLC [5,6,8,9,13].

Multivariate statistics were implemented to analyze the basic characteristics and differences of metabolites from the eight regions and quality control (QC) samples. HCA (Figure 2A) revealed differences in metabolite contents and clusters. We found that the fruits of AH were rich in organic acids and phytohormones, YN was rich in flavonoids and others, SC was rich in steroids and derivatives and GZ was rich in terpene. From the clustering results (Figure 2C), we observed that AH, CQ and ZJ were clustered together, SD, GZ, SC were clustered together and HB, QC, ZJ1 and SD3 were clustered together. YN and other groups were classified as one group. PCA, a standard method of data preprocessing, was performed to reveal the internal structure of multiple variables. Quality control (QC) samples are special samples that are formed by mixing all *C. speciosa* fruit sample extracts. From Figure 2D, it can be seen that QC samples are closely distributed and even overlap near the coordinate axis origin, suggesting that their metabolite content was close, the stability of the detection machine was good and the experimental results were reliable, accurate and repeatable [33]. The results show that the eight samples from the eight regions were compartmentalized into two groups, which indicates that each group had similar metabolic profiles. Based on PCA analysis (Figure 2D and Figure S1), Group 1 consisted of seven different regions samples: CQ, GZ, HB, SD, ZJ, SC and AH. The metabolic profiles of CQ, GZ, HB, ZJ, SC and SD were so similar that the points distributed together and even overlapped each other. Group 2 consisted of YN and was clearly different from the other producing areas.

The PCA and HCA results indicate that the metabolic profiles of YN differed from the others, and each origin has its own metabolic profile. Meanwhile, many previously unreported components were found.

### 3.3. Identified Effective Metabolites in the TCMSP Database

Previous studies of *C. speciosa* fruits primarily focused on the research of pharmacology and biological activities, such as antioxidant activity [4], anti-inflammatory effects [4], antiglucosidase activity [34], anticancer activity [2], antiviral activity [4], antitumor activity [35], antibacterial activity [6] and antipathogenic bacteria [36]. For *C. speciosa* fruits, although the Pharmacopoeia of the People’s Republic of China designated two legal triterpenes, it does not mean that *C. speciosa* has only two effective components. 

Therefore, we further searched and matched 974 metabolites in the TCMSP database [18] to determine the effective components related to disease resistance and drug targets. Ultimately, we matched 450 metabolites (Table S2), 326 out of 450 compounds related to disease resistance and 336 compounds related to drug targets. Among the 326 metabolites related to disease resistance (Figure S2), 91 flavonoids, 38 terpenes, 28 alkaloids, 26 phenols, 25 phenylpropanoids, 17 amino acids and derivatives, 13 coumarins, 12 lipids, 12 steroids and derivatives, 10 lignans, 9 vitamins and derivatives, 8 carbohydrates and alcohols, 7 organic acids and derivatives, 6 quinones, 5 benzene and substituted derivatives, 4 nucleotide and derivates, 1 xanthone, 1 phytohormone and 13 other metabolites were included. In the same way, among the 336 metabolites related to drug targets (Figure S2), 93 flavonoids, 40 terpenes, 32 alkaloids, 27 phenols, 25 phenylpropanoids, 17 amino acid and derivatives, 13 coumarins, 12 lipids, 12 steroids and derivatives, 10 lignans, 9 vitamins and derivatives, 8 carbohydrates and alcohols, 7 organic acids and derivatives, 6 quinones, 5 benzene and substituted derivatives, 4 nucleotide and derivates, 2 phytohormones, 1 xanthone and 13 other metabolites were contained. From Figure S2, we can see that YN had the highest relative content in disease resistance and drug targets, mainly enriched in flavonoids. 

**Figure 3:**
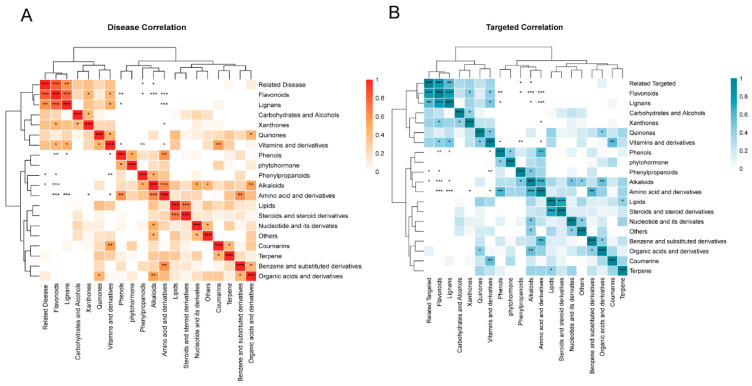
(**A**) Pearson correlation analysis between 19 compound classes and 326 related disease metabolites. (**B**) Pearson correlation analysis between 19 compound classes and 326 related targeted metabolites. * Significant correlation between different classes and diseases or targets, *p* < 0.05. ** Significant correlation between different classes and diseases or targets, *p* < 0.01. *** Significant correlation between different classes and diseases or targets, *p* < 0.001.

Then, we performed a Pearson correlation analysis and drew a clustering heat map to illustrate the bioactive ingredients related to disease resistance and drug targets, as shown in Figure 3. We found that flavonoids not only had the highest content but also had the most significant correlation among all metabolites related to diseases or targets. Furthermore, lignans, phenylpropanoids and alkaloids were also significantly related to diseases or targets. To further search for potential key active metabolites, we used the parameters oral bioavailability (OB) ≥ 30% and drug likeness (DL) ≥ 0.18 as the selection criteria [18,37]. The results show that 101 out of the 450 components were identified (Table S2). Among the 101 substances, 87 were most probably related to disease resistance, and 88 were very likely to be related to targets. Of the 87 disease-related metabolites, 37 flavonoids were the potentially primary disease-resistant components, but 50 nonflavonoids also potentially positively affected human health. These results largely enrich our understanding of the cardinal active components of *C. speciosa* fruits in the treatment of human diseases and drug targets. Meanwhile, they also provide clear evidence that *C. speciosa* fruits had not only two effective active ingredients but also had more active metabolites.

### 3.4. Correlation between Climatic, Major and Differential Metabolites in Different Regions

We found that there was a strong negative correlation between latitude and longitude and the content of flavonoid metabolites. The higher the latitude (°N), the lower the flavonoid content. The same situation also occurred above the longitude (°E). However, there was no strong correlation between metabolites and climate. The correlation analysis results were listed in Table 2.

To find the differential metabolites between the different producing areas, we carried out OPLS-DA analysis. The OPLS-DA analysis results were listed in Table S3 and Figure S3. According to the selection criteria, VIP > 1 and FC < 0.5 or >2, 548 out of the 974 differential metabolites were sorted. 

**Figure 4:**
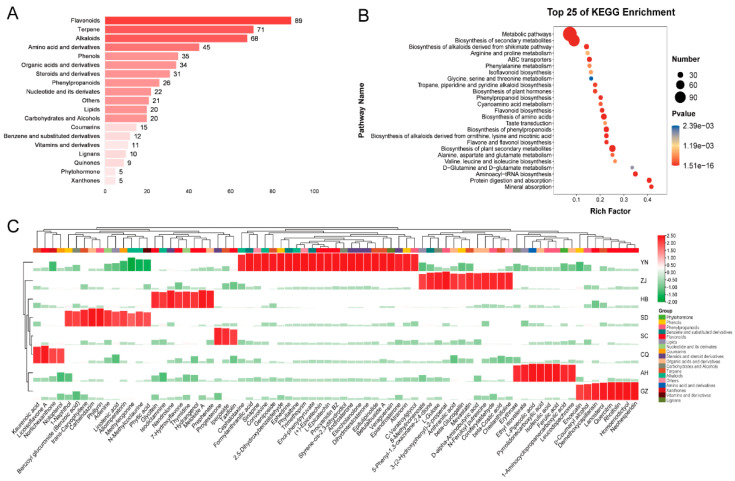
Analysis of 548 differential metabolites. (**A**) Classification of 548 differential metabolites of *C. speciosa* (**B**) KEGG enrichment analysis of 548 differential metabolites and the top 25 pathways. (**C**) Potential marker metabolites selected to visualize the marker metabolites of each region.

According to the number of classes from high to low (Figure 4A), there were 89 flavonoids, 71 terpenoids, 68 alkaloids, 45 amino acid and derivatives, 35 phenols, 34 organic acids and derivatives, 31 steroids and derivatives, 26 phenylpropanoids, 22 nucleotide and derivates, 20 lipids, 20 carbohydrates and alcohols, 15 coumarins, 12 benzene and substituted derivatives, 11 vitamins and derivatives, 10 lignans, 9 quinones, 5 phytohormones, 5 xanthones and 21 other metabolites. We drew a heat map of the 548 differential metabolites and selected every region with significantly more compounds to visualize a new regional biomarker map, as shown in Figure 4C. These results indicate that each producing area had its potential unique marker metabolite. In this study, YN had the largest number of potential marker metabolites, including formylanthranilic acid, sinapine, convolvine, geniposide, 2,5-dihydroxybenzaldehyde, ephedrine, phyllalbine, trimethoprim, enol-phenylpyruvate, (+)-epicatechin, (−)-epicatechin, procyanidin B2, atyrene-cis-2,3-dihydrodiol, androsterone, etiocholanolone, dihydrotestosterone, epitulipinolide, betulalbuside A, veraguensin, epiandrosterone, atranorin and C-veratroylglycol. SC had the least potential marker metabolites, ipecoside, progesterone and taxifolin. 

After the 548 differential metabolites were investigated with KEGG enrichment analysis, we obtained the 46 significant enrichment pathways of which *p* < 0.05 and selected the top 25 pathways to plot by ranking the *p*-values from low to high, as shown in Figure 4B. We were able to visualize the biosynthesis of flavonoids (ko00941, ko00944, ko00943), biosynthesis of amino acids (ko01230), biosynthesis of phenylpropanoids (ko01061) and biosynthesis of alkaloids derived from ornithine, lysine and nicotinic acid (ko01064) and so on. These results indicate that the differential metabolite analysis results were reliable, the metabolic profiles of each region were clear and flavonoids were the most dominant differential metabolites.

### 3.5. Determination of Core Region and Comparison with Other Regions

**Figure 5:**
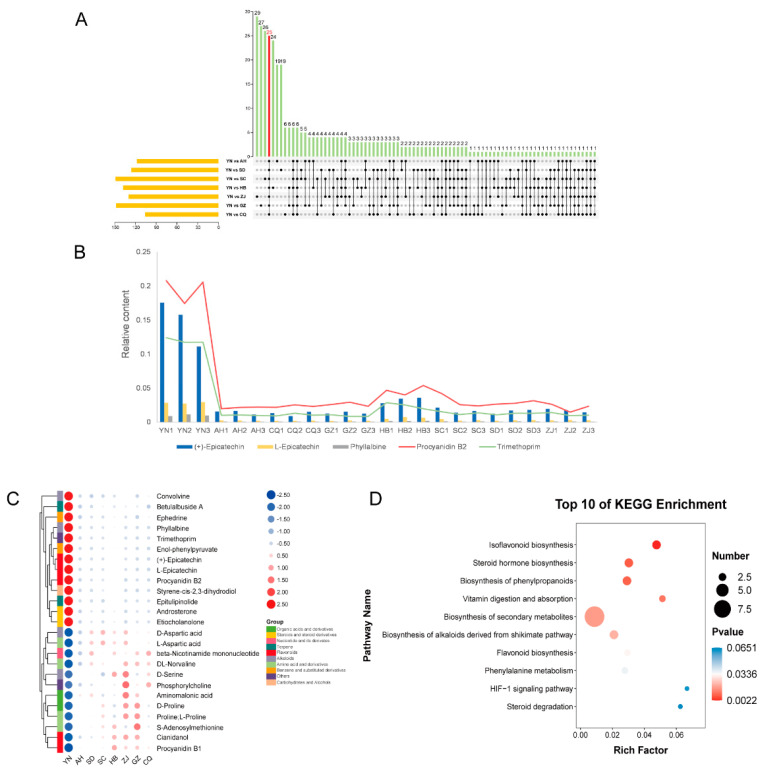
Comparison of differential metabolites between YN and other regions. (**A**) Upset diagram was applied to depict commonly expressed metabolites. (**B**) Five metabolites with the highest content in the shared metabolites. (**C**) Heat map of 25 common metabolites. (**D**) KEGG enrichment analysis of differential metabolites with higher content in YN.

The PCA, HCA analysis and permutation tests results (Figure 2C,D and Figure S4) show that YN was significantly different from the other producing areas. This means that YN could be compared with the other groups as a critical group. An upset diagram was applied to depict commonly expressed metabolites among YN vs. AH, YN vs. SD, YN vs. SC, YN vs. HB, YN vs. ZJ, YN vs. GZ, YN vs. CQ (Figure 5A). Ultimately, 25 common differential metabolites were selected as the key metabolites of YN (Figure 5C). Then, to better distinguish the differences between them, we drew a cluster heat map, which indicated that 13 substances were enriched in the YN-producing area: androsterone, betulalbuside A, convolvine, enol-phenylpyruvate, ephedrine, (+)-epicatechin, epitulipinolide, etiocholanolone, (−)-epicatechin, phyllalbine, procyanidin B2, styrene-cis-2,3-dihydrodiol, trimethoprim. The contents of 12 substances were minimal in the YN region: aminomalonic acid, beta-nicotinamide mononucleotide, cianidanol, D-aspartic acid, D-proline, D-serine, DL-norvaline, L-aspartic acid, phosphorylcholine, procyanidin B1, Proline, S-adenosylmethionine (Figure 5C). Five of the twenty-five compounds were found in YN, with exceptionally high content, such as (+)-epicatechin, (−)-epicatechin, phyllalbine, procyanidin B2, trimethoprim (Figure 5B). These five substances could also be regarded as high-content metabolites in other producing areas. 

Zheng et al. [9] investigated the metabolic profiling of *C. speciosa* fruit extracts from four producing areas in China. The results showed that Yunnan had the highest total flavonoid and total polyphenol content and antioxidant and α-glucosidase inhibitory activity. YN is located in the southernmost of the eight sample regions, with sufficient sunshine and abundant heat, which probably leads to the high content of total flavonoids [9]. Meanwhile, we found that the contents of three flavonoids, (+)-epicatechin, (−)-epicatechin and procyanidin B2, were high. (−)-Epicatechin is an antioxidant flavonoid and an enantiomer of a (+)-epicatechin. Procyanidin B2 is composed of two (−)-epicatechin molecules and also has antioxidant activity. These findings explain why YN has higher antioxidant activity [9]. Furthermore, we found abundant trimethoprim had antibacterial and antimalarial properties, which is a powerful supplement to Luo’s antibacterial experiment of *C. speciosa* fruits [5].

We screened the metabolites of YN with higher content than the other places and performed KEGG enrichment analysis, as shown in Figure 5D. On the basis of the KEGG enrichment results, 10 metabolic pathways were enriched, and these key metabolites clearly described which pathways were enriched in YN. These results suggest that the biosynthesis of isoflavonoids and flavonoids, biosynthesis of steroid hormones, biosynthesis of phenylpropanoids and biosynthesis of alkaloids derived from the shikimate pathway were the main enrichment pathways in the YN regions.

## 4. Conclusions

We collected fresh fruits from eight geographical regions in the present study and recorded detailed geographical locations and coordinates. Based on the widely targeted metabolomics analysis of UHPLC-QqQ-MS/MS, *C. speciosa* fruits of eight regions were systematically identified and compared. The experimental results show that *C. speciosa* fruits from the eight regions differed in their metabolite contents. Ecological factors usually influence the composition of metabolites, and this influence was particularly reflected in YN. We found that there was a strong negative correlation between latitude and longitude and the content of flavonoids metabolites. Each producing area had more or less its own biomarker metabolite, and YN had the most. This is the first study to combine *C. speciosa* with the TCMSP database, and important information such as data source is sorted and summarized in Table S2. Based on OB ≥ 30% and DL ≥ 0.18 as the selection criteria, a total of 101 metabolites were identified as key active compounds. These results largely enrich our understanding of the cardinal active components of *C. speciosa* fruits in the treatment of human diseases and drug targets. These results could assist researchers in purposefully selecting regions and meeting the requirements for breeding or extracting natural products.

## Figures and Tables

**Table 1:** Fruit dimensions of *C. speciosa*.

Area	Length (cm)	Long Diameter (cm)	Short Diameter (cm)	Single Weight (g)
YN	7.73 ± 0.35 ^bc^	6.54 ± 0.18 ^bc^	6.09 ± 0.34 ^cd^	184.53 ± 16.24 ^bc^
HB	10.65 ± 0.36 ^A^	6.88 ± 0.13 ^bc^	6.44 ± 0.25 ^cd^	214.97 ± 13.69 ^b^
AH	7.02 ± 0.6 ^c^	5.25 ± 0.34 ^d^	4.97 ± 0.39 ^e^	96 ± 2.16 ^d^
SD	7.07 ± 0.27 ^c^	6.37 ± 0.21 ^bc^	6.03 ± 0.32 ^cd^	146.67 ± 15.93 ^cd^
SC	10.91 ± 0.36 ^A^	7.89 ± 0.28 ^A^	7.86 ± 0.25 ^A^	334.07 ± 29.73 ^A^
ZJ	8.25 ± 0.6 ^b^	7.17 ± 0.66 ^ab^	6.74 ± 0.47 ^bc^	204.6 ± 44.89 ^b^
CQ	8.45 ± 0.31 ^b^	6.1 ± 0.25 ^c^	5.72 ± 0.4 ^de^	141.13 ± 4.53 ^cd^
GZ	10.66 ± 0.77 ^A^	7.74 ± 0.52 ^A^	7.23 ± 0.35 ^AB^	283.77 ± 31.92 ^AB^

^A^ Significant difference from different areas in fruit dimensions, *p* < 0.01. ^a^ Significant difference from different areas in fruit dimensions, *p* < 0.05. Data that are significant different (*p* < 0.05) are denoted with different letters.

**Table 2:** Correlation analysis between climatic and major metabolite classes in *C. speciosa*.

	Amino Acid	Benzene	Phenols	Nucleotides	Flavonoids	Alkaloids	Sugar Alcohols	Terpenoids	Vitamins	Organic Acids	Lipids
Altitude(m)	−0.540	−0.399	−0.265	−0.377	0.655	−0.125	0.163	−0.133	0.585	−0.550	0.084
Average temperature (°C)	−0.419	−0.108	−0.151	−0.581	0.401	0.053	0.020	−0.247	0.499	−0.183	0.116
Annual rainfall (mm)	−0.560	−0.235	−0.353	−0.625	0.568	−0.321	0.049	−0.464	0.403	−0.028	−0.359
Lighting hours (h)	−0.080	0.023	0.105	0.183	−0.035	−0.123	0.179	−0.180	−0.243	0.037	−0.262
Longitude (°E)	0.749	0.482	0.303	0.448	−0.804	0.148	−0.429	0.127	−0.489	0.630	−0.163
Latitude (°N)	0.714	0.406	0.498	0.637	−0.810	0.275	−0.073	0.332	−0.519	0.322	0.135

## Data Availability

Raw data file will be uploaded following article publication. https://figshare.com/s/6373a3cebd1dcf45b42d, accessed on 28 February 2022.

## Supplementary Materials

The following supporting information can be downloaded at: https://www.mdpi.com/article/10.3390/foods11071019/s1. Table S1. List of the 974 metabolites, Table S2. TCMSP database list, Table S3. OPLS-DA model of the 28 groups. Figure S1. PCA loading plot. Figure S2. Relative content of the 326 related diseases, 336 related target metabolites and 19 classes. Figure S3. Comparison of OPLS-DA in 28 groups. Figure S4. YN vs. others permutation tests.
