# Supplementary figures and images for: Comparative Metabolomics Study of Chaenomeles speciosa (Sweet) Nakai from Different Geographical Regions
Source: Foods. 2022 Mar 31;11(7):1019. doi: 10.3390/foods11071019 (PMC8997580; doi:10.3390/foods11071019)

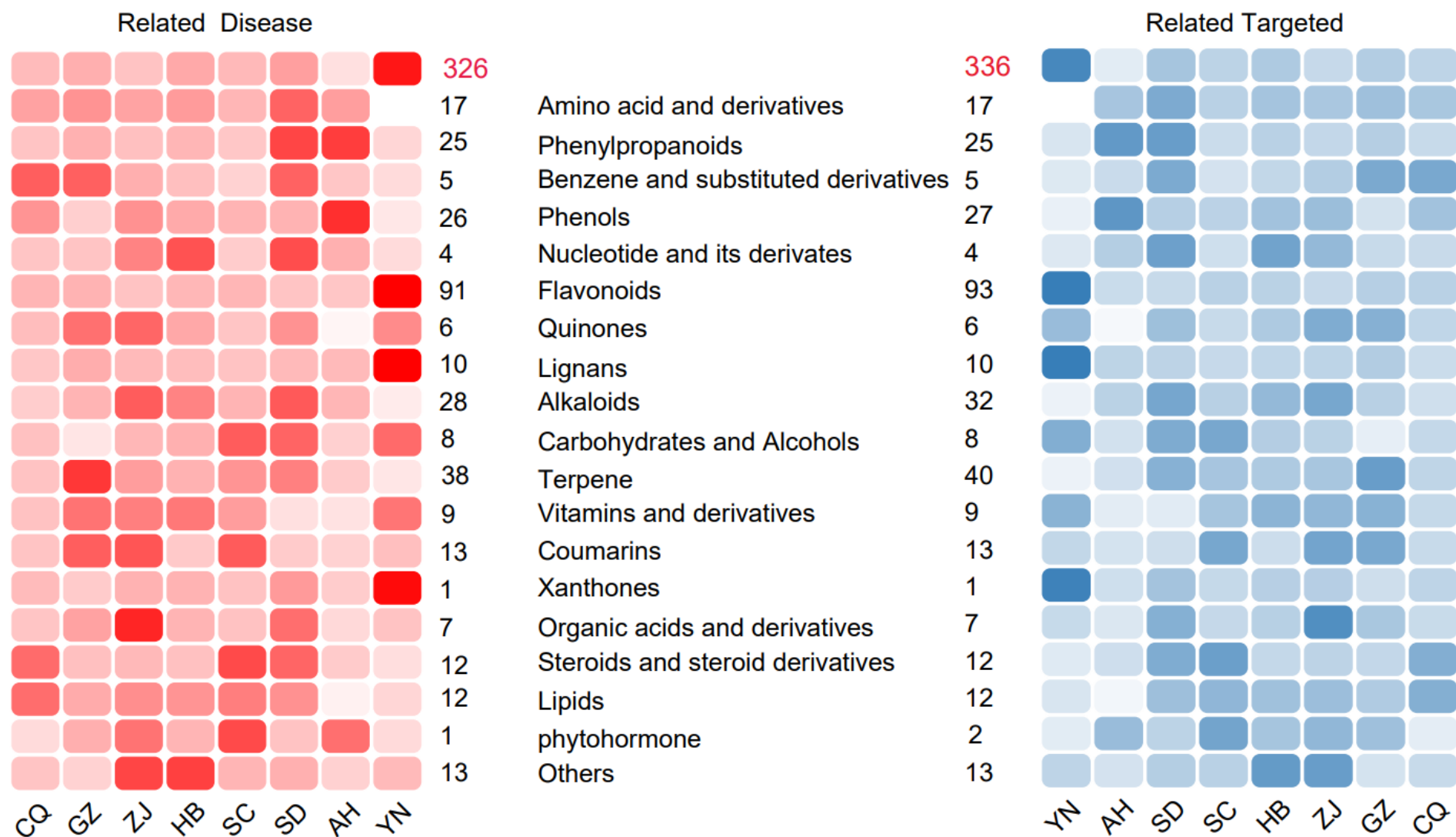

Figure S1. 326 related disease and 336 related targeted metabolites and 19 classes relative content

Supplement: Supplementary file 1 [file foods-11-01019-s001.zip › Figure S1.pdf]

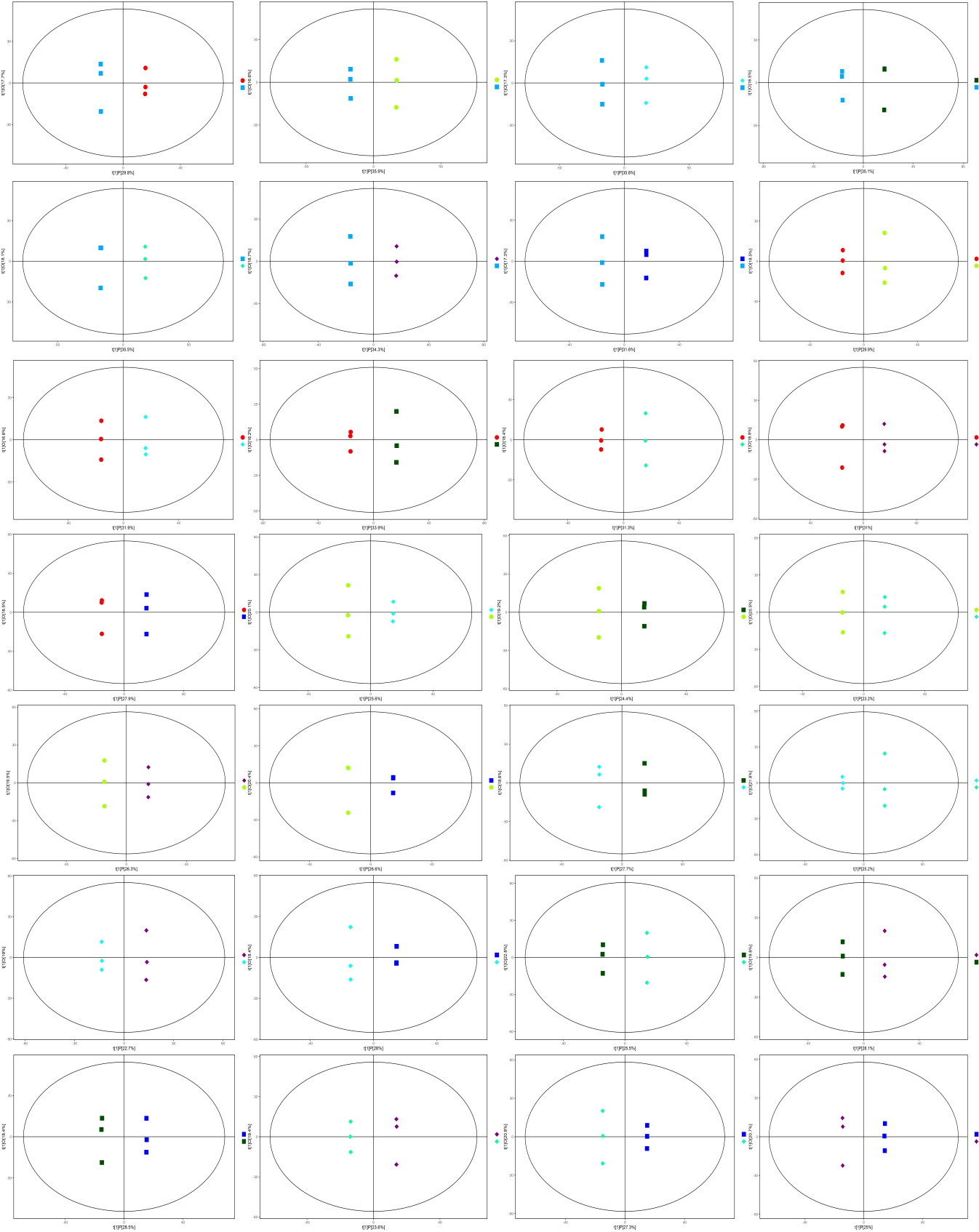

Figure S2. Comparison of OPLS-DA in 28 groups.

Supplement: Supplementary file 1 [file foods-11-01019-s001.zip › Figure S2.pdf]

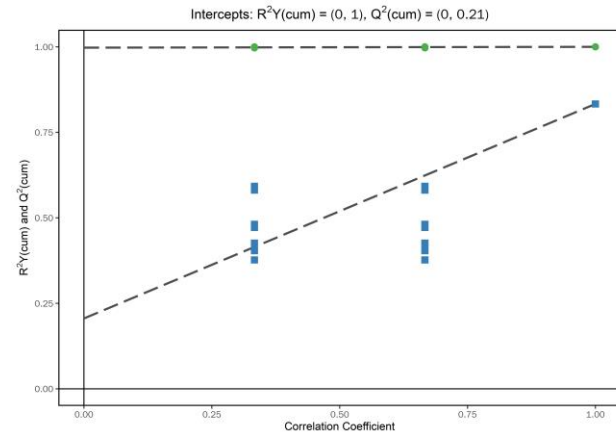

YN VS AH

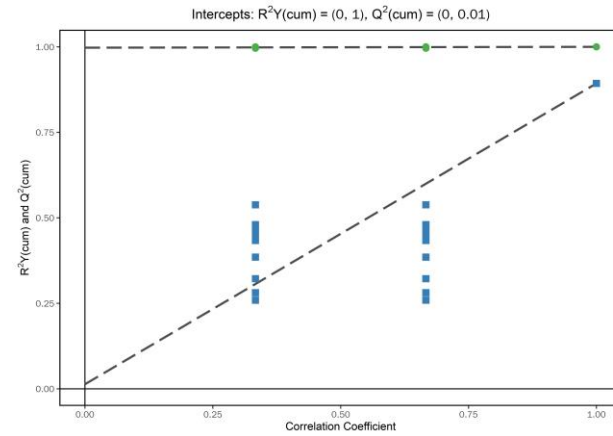

YN VS HB

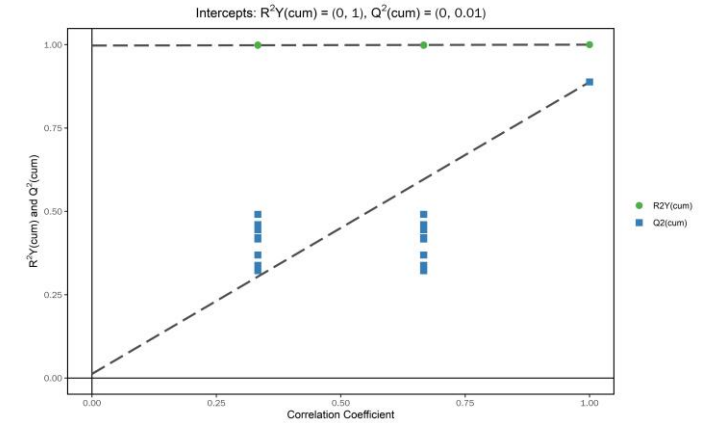

YN VS SD

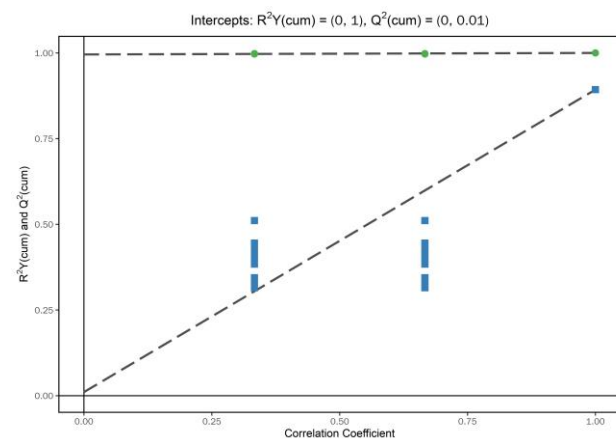

YN VS SC

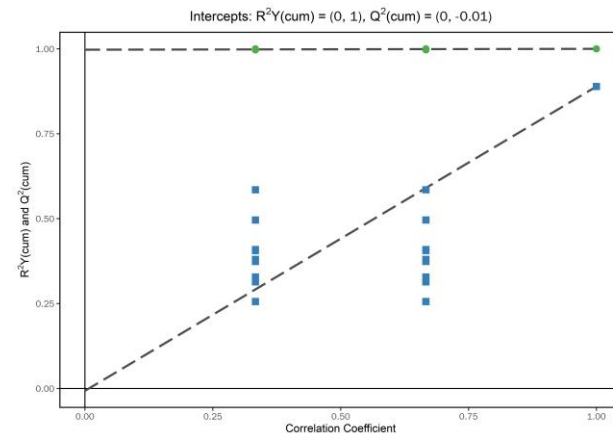

YN VS ZJ

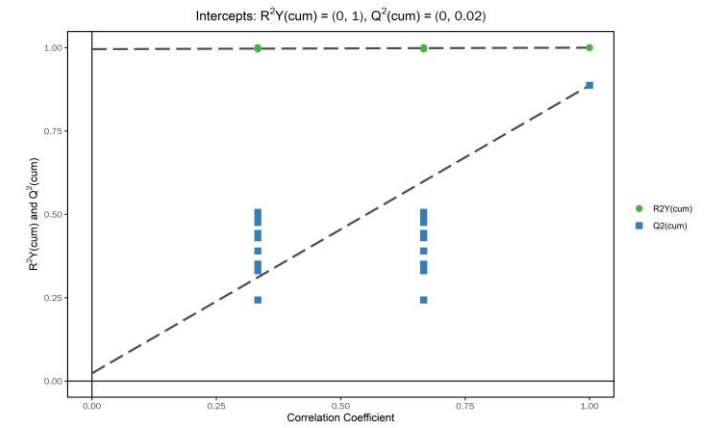

YN VS GZ

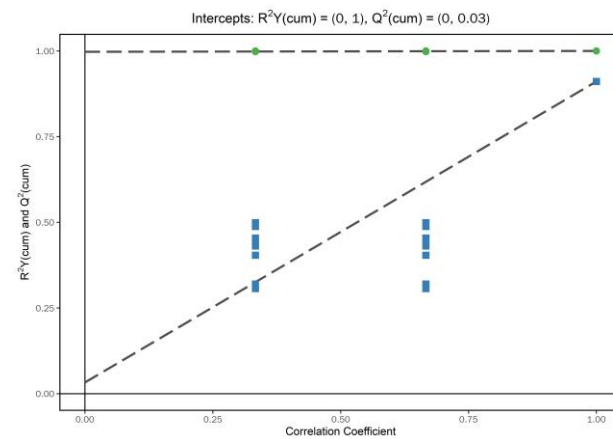

YN VS CQ

Figure S3. YN vs others permutation tests

Supplement: Supplementary file 1 [file foods-11-01019-s001.zip › Figure S3.pdf]

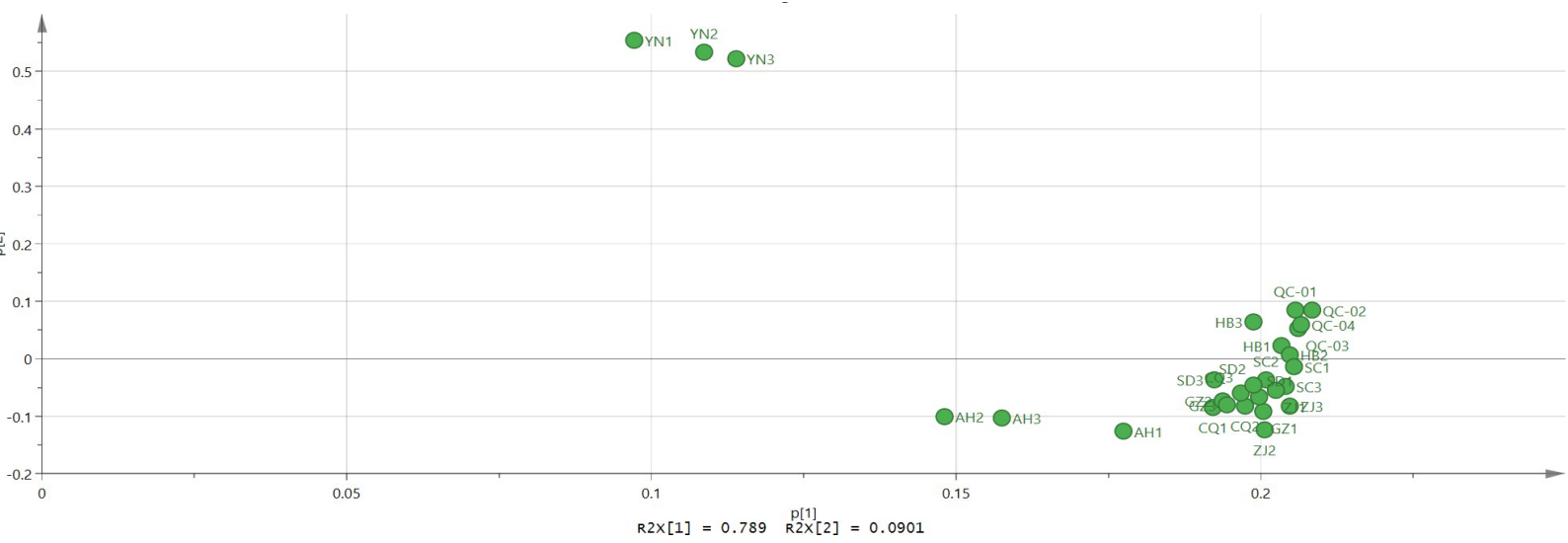

Figure S4 PCA loading plot

Supplement: Supplementary file 1 [file foods-11-01019-s001.zip › Figure S4.pdf]
